# Characterizing the spatial mismatch between intimate partner violence related healthcare services and arrests in Miami-Dade County, Florida

**DOI:** 10.1186/s12889-018-5985-5

**Published:** 2018-08-31

**Authors:** Jessica Williams, Nick Petersen, Justin Stoler

**Affiliations:** 1 School of Nursing, University of North Carolina at Chapel Hill, 5004 Carrington Hall, Campus Box 7460, Chapel Hill, North Carolina 27599-7460 USA; 2 Department of Sociology, University of Miami, 5202 University Drive, Merrick Building, Room 122G, Coral Gables, Florida USA; 3 Department of Geography, University of Miami, 1300 Campo Sano Ave, Coral Gables, FL 33146 USA; 4 Department of Public Health Sciences, Miller School of Medicine, University of Miami, 1120 NW 14th St, Miami, FL 33136 USA

**Keywords:** Domestic violence, Health services accessibility, Healthcare disparities, Geographic information systems

## Abstract

**Background:**

Routine screening and intervention for intimate partner violence (IPV) in healthcare settings constitutes an important secondary prevention strategy for identifying individuals experiencing IPV early and connecting them with appropriate services. Considerable variation in available IPV-related healthcare services exists and interventions are needed to improve the quality of these services. One way to prioritize intervention efforts is by examining the level of services provided in communities most at risk relative to local incidence or prevalence of IPV. To inform future interventions, this study examined the spatial relationship between IPV-related healthcare services and IPV arrests in Miami-Dade County, Florida, and identified predictors of the observed spatial mismatch.

**Methods:**

Survey data collected in 2014 from 278 health facilities pertaining to IPV services were geocoded, computed into a density layer, and aggregated at the census tract level to create a population-based normalized comprehensiveness score (NCS) as a proxy for IPV-related healthcare resources. IPV arrests from 2011 to 2015, collected from the county court, were geocoded and summarized by census tracts to serve as a proxy for IPV prevalence. These measures were combined into a resource disparity score (RDS) that compared relative service density to relative arrest rates, where positive RDS represented over-resourced neighborhoods and negative RDS corresponded to under-resourced neighborhoods. We used correlation analyses and a two-phase spatial modeling approach to evaluate correlates of NCS and RDS.

**Results:**

A spatial lag model did not yield an association between NCS and IPV arrests, demonstrating a spatial mismatch, which we visualized using a Geographic Information System (GIS). A spatial error model revealed that the percentage of white non-Hispanic residents was positively associated with RDS, while percent black non-Hispanic, median age, ethnic heterogeneity, and economic disadvantage were negatively associated with RDS.

**Conclusions:**

These findings underscore the need to further evaluate the adequacy of IPV-related healthcare resources for secondary prevention relative to local IPV arrest rates, particularly within economically disadvantaged neighborhoods. Our approach demonstrates the utility of GIS for identifying potential priority regions for IPV prevention efforts and resource allocation.

## Background

The relationship between intimate partner violence (IPV) victimization and adverse health outcomes is well established [1–4]. These outcomes not only include acute injury as a result of the violence, but also chronic, long-term physical and mental health consequences. Victims of IPV are more likely to seek health services compared to those with no history of victimization [5, 6] and health care seeking is often driven by the availability of resources within a community [7, 8]. As such, it is important to understand how resources for addressing IPV within healthcare settings are distributed given that healthcare visits provide an opportune time to identify patients with a history of IPV and intervene.

Over the last decade, there has been an unprecedented increase in support for the integration of routine screening and intervention for IPV into healthcare settings in the United States (U.S.) and around the world. Numerous government and professional organizations have developed recommendation statements and guidelines for routine screening and intervention for IPV [9–12]. These services constitute an important secondary prevention strategy for identifying individuals experiencing IPV early and connecting them with appropriate services to mitigate further adverse consequences.

Recent research demonstrates a general awareness among health providers of their role in identifying and responding to IPV, but with considerable variation in the level of services provided. Through a survey conducted with 288 healthcare facilities in Miami-Dade County (MDC), Florida, Williams and colleagues [13] found that a majority of facilities (78.1%) provided some type of screening and/or intervention for IPV, yet only 35.3% offered comprehensive IPV services following evidence-based recommendations [14].

It is important to continue exploring this variation in available IPV-related healthcare services because the degree of service availability or comprehensiveness can produce variable results on patient outcomes. For example, some IPV prevention resources have been found to be related to an *increase* in partner homicide, as interventions that insufficiently reduce exposure can lead to retaliation behavior by the aggressor [15]. There is a need for interventions to promote evidence-based IPV screening and response services within healthcare facilities. In addition, there is a need for methods to target healthcare facilities most in need of these types of interventions. One way to prioritize resources for healthcare facilities is by examining the level of services provided in communities most at risk relative to local incidence or prevalence of IPV.

Geographic Information Systems (GIS) are increasingly being used as a framework for explicitly analyzing the spatial factors associated with IPV rates and experiences [16]. Spatial frameworks have been applied to study drivers of IPV, such as concentrated poverty [17], neighborhood disorder [18, 19] and access to IPV resources [20, 21]. These studies find higher rates of IPV incidence in low-income and minority neighborhoods as well as those with less access to resources. Unequitable distribution of health care resources can result from numerous factors, including political drivers (e.g., allocating resources to areas deemed preferable by local leadership) and financial drivers (e.g., designating services to areas based on profit rather that public health need). Prior research has shown that IPV-related healthcare services are geographically concentrated due to institutional processes such as zoning, and social processes such as residential segregation [21]. As a result, IPV resources are often less concentrated in economically disadvantaged and minority communities [21].

Despite research showing that minority and low-income communities often have *both* higher rates of IPV [22, 23] and fewer IPV-related resources [21], these topics remain disconnected in the literature. To the best of our knowledge, no previous studies have evaluated the availability of IPV-related healthcare services relative to IPV arrests as a means for evaluating the extent of spatial concordance between IPV-related need and resources, and what factors are associated with any observed spatial mismatch. This is an important omission in the literature because we do not currently know whether IPV resources are being directed to the communities that need them most, although insights from the previous research suggest that there is likely a spatial mismatch. The current study attempts to fill this gap by comparing a measure of IPV-related healthcare service comprehensiveness to IPV arrest rates in MDC, Florida, utilizing these variables as proxies for IPV-related healthcare service availability and IPV prevalence. We evaluate the relationships between these measures by testing three interrelated hypotheses:

1. Census tract-level IPV arrest rates will be associated with local comprehensiveness of IPV-related healthcare services, quantified as a *normalized comprehensiveness score* (NCS), after controlling for socio-demographic factors;
2. Regardless of the strength or direction of this association, we hypothesize that there will be some spatial mismatch between NCS and IPV arrest rates, with some areas being over- and under-resourced relative to local IPV arrest rates;
3. This spatial mismatch, quantified as a *resource disparity score* (RDS), will be associated with indicators of racial/ethnic composition and concentrated disadvantage. More specifically, we hypothesize that whiter and wealthier neighborhoods will be over-resourced, while areas with a larger racial/ethnic minority and economically disadvantaged population will be under-resourced.

This spatial approach will help identify neighborhoods that have higher IPV arrest rates yet are relatively under-resourced with respect to available IPV-related healthcare services. Doing so will also highlight any socio-demographic characteristics associated with high disparities between IPV arrests and healthcare services so that relevant interventions and resources can be better targeted to communities in need.

## Methods

### Study site

MDC is an important research site to investigate the spatial relationship between IPV-related healthcare services and arrests given its racial, ethnic, and socioeconomic diversity. With over 2.7 million residents, MDC is the seventh-largest US county by population [24]. The county’s geographic location in South Florida and its diverse Hispanic majority population have earned MDC a reputation as the “Gateway of the Americas.” MDC has a legacy of displacing the black non-Hispanic (hereafter referred to as black) population to accommodate urban development projects, while the wealthier white non-Hispanic (hereafter referred to as white) population has tended to settle along coastal and beach areas. The resulting trends yield distinct geographic patterns of residential segregation and economic inequality characterized by the “new geography of inequality” [25].

MDC’s demography and stark segregation made it something of an outlier throughout much of the twentieth century. However, new research has shown that as American cities diversify, they also tend to become more segregated [26, 27], increasingly resembling the pattern produced by MDC’s waves of immigration and racially-driven politics. MDC’s rich diversity and global economy now position it as a prototypical twenty-first century metropolis [28], and thus a representative model for studying violence in an American city.

### Data

This study overlaid data from health-care providers, law enforcement agencies compiled by the courts, and the U.S. Census in a GIS to examine the relationship between IPV-related health services and arrests in MDC. Information on IPV-related policies and practices were obtained from phone surveys of health-care providers [13], while IPV arrest rates come from the MDC Clerk of the Court’s office. These data sources were aggregated to the census tract level and combined with a subset of neighborhood demographic variables from the 2010 census for analysis. Our analytic sample of 503 census tracts excluded tracts with low populations outside of MDC’s urban development boundary, as well as those containing Miami International Airport, Miami-Opa Locka Executive Airport, and Miami Executive Airport.

### Normalized comprehensiveness score (NCS)

The first dependent variable was a census tract’s mean NCS of IPV-related healthcare services. The NCS measure, developed through previous work by two of the authors (JW and JS), was derived from phone surveys conducted in 2014 of 278 randomly selected primary care (*n* = 72), obstetrics/gynecology (*n* = 93), pediatric (*n* = 106), and emergency department (*n* = 17) facilities out of a county-wide sampling frame of 1208 [13]. The NCS represents the geographic density of a screening comprehensiveness index that classified facilities as offering high, medium, low, or no IPV screening comprehensiveness [full methodology is reported elsewhere [13, 21]]. A Gaussian kernel density surface was generated from the index values at each healthcare facility location in a GIS using a 1 km kernel density and inverse-distance weighting, and then the mean value of all pixels within a census tract was extracted and normalized by population. We thus conceptualized NCS as a proxy measure of IPV-related healthcare service availability.

### Resource disparity score (RDS)

Our second dependent variable, developed by the authors specifically for this study, measured the mismatch between IPV-related healthcare services and IPV arrests. We conceptualized this mismatch as the *resource disparity score* (RDS), which we calculated by subtracting the *Z*-score of a census tract’s IPV arrest rate from the NCS *Z*-score. Thus, tracts with a positive RDS can be considered relatively over-resourced with respect to IPV-related health service density, while tracts with a negative RDS can be considered relatively under-resourced. Tracts with the lowest RDS are not necessarily those with the worst IPV arrest rates, but rather those with the most extreme disparity between resources and arrests. The tracts of highest priority for anti-violence interventions would, therefore, be those with the highest arrest rates and the lowest RDS.

### IPV arrest rates

IPV arrest data, which we obtained from the MDC Clerk of Courts through a public records request, contains every misdemeanor IPV arrest involving adults during the five-year period from January 1, 2011 to December 31, 2015. The MDC Clerk’s Office acts as a centralized data collection point by compiling arrest information from over 30 police departments across the county into a standardized format. IPV arrests were geocoded based on the arrest location and aggregated by census tract as an arrest rate per 1000 residents.

We used the IPV arrest rate as a proxy for IPV incidents because incident data on IPV victimization is not systematically collected in MDC. We believe this is a reasonable proxy since the majority of MDC police agencies have a mandatory or pro-arrest policy for IPV incidents. We also focused on misdemeanor IPV arrests because they constitute the majority of domestic violence cases. For example, Wooldredge and Thistlethwaite [29] found that felonies constitute only 7% of domestic violence cases in Ohio, and thus their analysis focused on misdemeanors. In addition, misdemeanor IPV cases are more systematically coded by the MDC Clerk’s Office. In MDC, misdemeanor IPV cases are sent to a specialized domestic violence court and, thus, are carefully tracked. Felony IPV cases, on the other hand, are prosecuted in the court’s general felony division and classified as assault, homicide, etc., making it difficult to determine which felony cases specifically involve IPV.

### Socio-demographic control variables

We controlled for a subset of neighborhood socio-demographic factors using data from the 2010 Census and American Community Survey (2006–2010) that may be related to NCS and RDS. We selected demographic characteristics that have been previously linked to disparities in access to health services [30, 31] and IPV prevalence [32, 33]. Neighborhood racial/ethnic composition is measured as the percentage of white, black, and Hispanic residents in each census tract. Additional races/ethnicities (e.g., Asian, Native Americans, etc.) were too small to power separate analyses, and thus were included in the reference category along with Hispanics. Racial and ethnic diversity is captured using the Herfindahl dissimilarity index. The Herfindahl index is computed as 1 minus the sum of squared proportions of each race/ethnicity, such that higher values indicate a higher degree of racial/ethnic diversity. We also included median age to control for the local age structure. To capture variability in the housing and employment markets, we included median gross rent and the percentage of social security benefits recipients. Generally following Sampson et al. [34], we constructed a concentrated disadvantage index via principal components analysis using the following indicators (eigenvalues in parentheses): percent below poverty line (λ = 0.84), median house value (λ = − 0.80), median household income (λ = − 0.88), percent single family households (λ = 0.74), and percent receiving public assistance (λ = 0.58). In contrast to Sampson et al. [34], we do not include the percentage of Black residents in this index, instead measuring this variable separately. Higher values on the index indicate a greater degree of disadvantage.

### Statistical analysis

We began by calculating bivariate Pearson’s correlations and conducting exploratory, aspatial stepwise regression models using the two dependent variables (NCS and RDS) and the set of possible independent variables to assess potential multicollinearity and guide the multivariable spatial modeling process. Bivariate correlation analyses and stepwise models were performed using SPSS version 24 as an initial step (IBM Corp, Armonk, NY).

We then performed separate spatial regression models on NCS and RDS using data aggregated to 503 Miami-Dade census tracts. First, we built upon a spatial lag model of NCS from prior work, which observed that NCS was associated with ethnicity, age, median gross rent, and the percent of the population receiving Social Security benefits [21], by replicating the original model and then introducing IPV arrest rate. This model helped to establish whether there was a spatial mismatch between IPV arrests and IPV-related healthcare services. Second, we modeled RDS, using the same variable set used to model NCS in addition to new potential predictors such as the Herfindahl and concentrated disadvantage indices, to identify sociodemographic factors that were associated with any observed spatial mismatch.

Spatial regression analyses were performed in OpenGeoDa version 1.0.1 (University of Chicago, Chicago, IL) using spatial regression models estimated by maximum likelihood that adjust for spatial autocorrelation (i.e., the degree of spatial similarity or dissimilarity of nearby values) [35], with spatial weights constructed using first-order rook-contiguity (i.e., a neighborhood definition characterized by the sharing of some length of border). We relied on Lagrange multiplier diagnostic tests in OpenGeoDa, which indicated whether a spatial lag or spatial error model was more appropriate for modeling NCS and RDS [35, 36]. Lagrange multiplier diagnostic tests indicated that a spatial lag model was a better fit for estimating NCS, and that a spatial error model was a better fit for estimating RDS. The spatial lag model for NCS takes the basic form in Eq. 1:

1 $$ y=\rho Wy+ X\beta +\epsilon $$

where W is the spatial weights matrix, ρ measures the degree of spatial autocorrelation (between − 1 and 1), and thus *ρWy* measures the spatial dependence in y. The spatial error model for RDS takes the basic form shown in Eq. 2:

2 $$ y=\lambda W\mu + X\beta +\epsilon $$

where *λ* measures the degree of spatial autocorrelation of *μ*, a vector of spatially autocorrelated error terms, and thus *λWμ* measures spatial dependence in the error term. We employed an iterative model building process in OpenGeoDa, guided by the parsimony principle, in which we introduced individual terms and combinations of terms and strived to build models with the strongest explanatory power relative to other candidate models (i.e., minimizing the Akaike information criterion [AIC]) and with the fewest covariates possible. This approach sometimes resulted in the removal of terms theorized to be associated with a given outcome measure but helped to reduce multicollinearity. All associations were interpreted using a significance threshold of *α* = .05, and spatial data were managed in ArcGIS 10.3 (ESRI, Redlands, CA). The local institutional review board reviewed the study and determined that the data did not constitute human subject research.

## Results

### Descriptive statistics

**Fig. 1:**
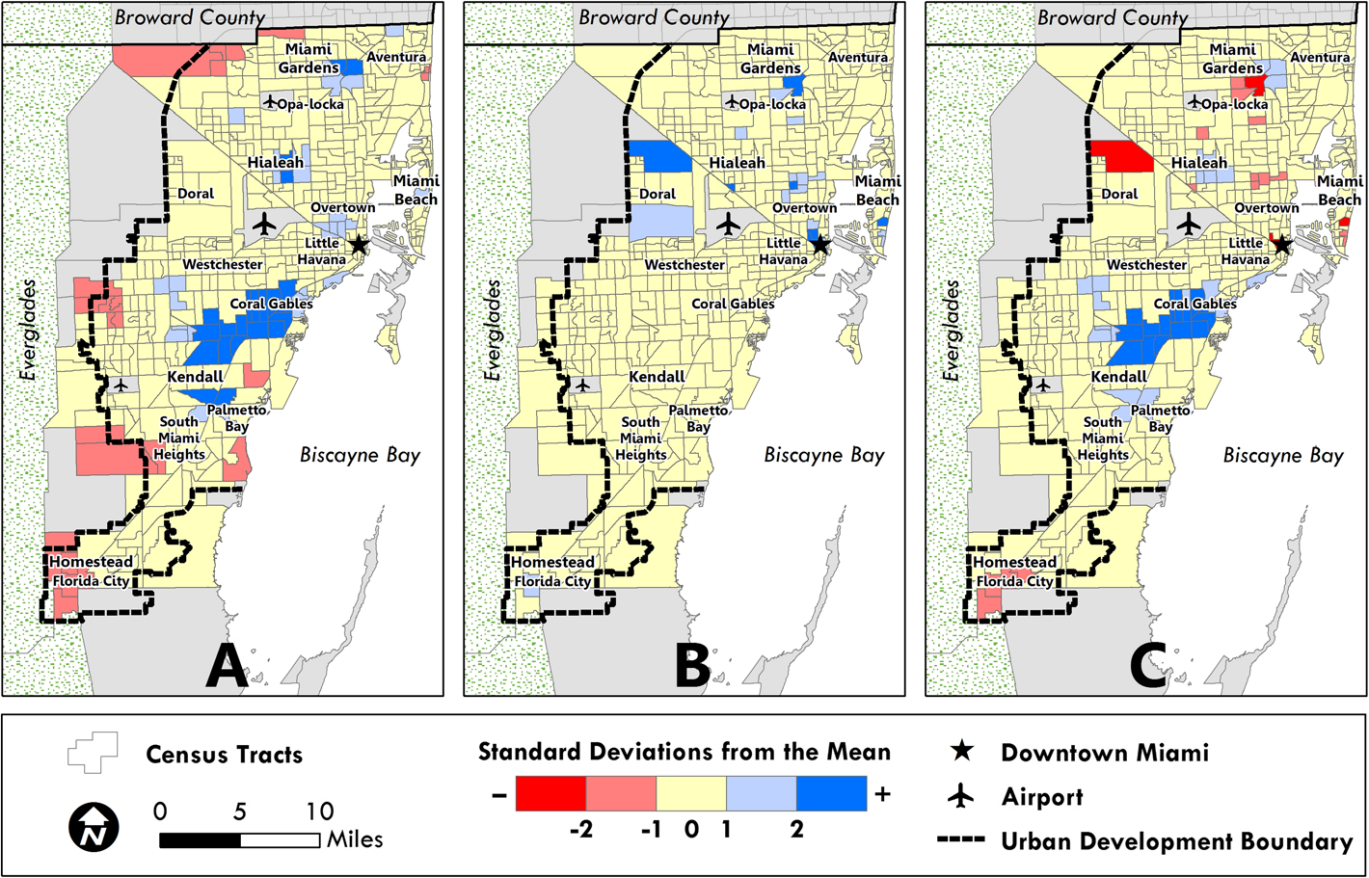
Distribution of Key IPV Indicators across 503 Census Tracts in Miami-Dade County, Florida. Distribution of **a** mean normalized comprehensiveness score (NCS), **b** intimate partner violence (IPV) arrest rate, and **c** IPV resource disparity score (RDS). Census tracts in light gray were excluded from analysis. *Source:* Figures generated by authors using ArcGIS 10.3 (ESRI, Redlands, CA)

Figure 1 displays standard deviation maps that highlight tracts with extreme values (above or below 2 standard deviations) in each panel. IPV-related healthcare services are primarily located in white neighborhoods to the southwest and south (panel A), while IPV arrests are mostly concentrated in black neighborhoods in the northern and central parts of the county (panel B). Given this spatial mismatch along racial lines, it is not surprising that white census tracts are over-resourced in terms of IPV-related healthcare services, while predominately black census tracts are typically under-resourced according to RDS (panel C).

Table 1 presents bivariate Pearson’s correlation coefficients for candidate predictors of NCS and RDS. The IPV arrest rate and NCS were not significantly correlated (*r* = −.002), suggesting that other factors aside from IPV arrests are related to where IPV screening and responses within healthcare facilities occur. To this point, NCS is correlated with several demographic variables included in Models 1–2. NCS is positively correlated with % white and % social security benefits, while NCS is negatively related to % black and median gross rent. RDS is positively correlated with % white and median gross rent, whereas RDS is negatively related to % black and the concentrated disadvantage index. The variables correlated with NCS and RDS provide preliminary information on factors that may be related to the geographic distribution of IPV-related healthcare services.

**Table 1:** IPV arrest rates and census tract characteristics for Miami-Dade County (*n* = 503), and Pearson’s correlations (*r*) with the normalized comprehensiveness score (NCS) and resource disparity score (RDS)

Characteristic	Tract Mean (SD)	Pearson’s Correlation (*r*) with NCS	Pearson’s Correlation (*r*) with RDS
IPV Arrest Rate (per 1000)	4.55 (9.06)	−.002	–
Black non-Hispanic (%)	16.86 (26.20)	−.042	−.203**
White non-Hispanic (%)	17.36 (17.23)	.108*	.155**
Hispanic (%)	63.16 (26.93)	−.036	.088*
Median Age (years)	38.78 (5.73)	.076	.068
No High School Diploma (%)	21.79 (13.51)	−.008	−.215**
Limited English Proficiency (%)	32.88 (18.01)	.002	−.148**
Median Gross Rent ($)	1170.97 (370.69)	−.123**	.097*
Median House Value ($)	298,677.93 (170,667.53)	.179**	.251**
Per Capita Income ($)	23,581.29 (14,932.11)	.089*	−.180**
Employed in Service Industry (%)	20.24 (11.03)	−.103*	−.340**
Renter-Occupied Housing Units (%)	38.46 (20.54)	.089*	−.135**
Population Below Poverty Line (%)	17.30 (12.39)	.013	−.283**
Receives Social Security Benefits (%)	26.67 (11.22)	.129**	.052
Female-Headed Households (%)	18.81 (8.29)	−.087	−.150**
Mobile Home Housing Units (%)	1.89 (8.82)	−.088*	−.188**
Housing Units with No Automobile (%)	11.39 (11.80)	.043	−.187**
Ethnic Heterogeneity Index	.36 (.20)	.027	−.023
Concentrated Disadvantage Index	.06 (.73)	−.091*	−.257**

*P < .05; **P < .01

*IPV* intimate partner violence, *NCS* normalized comprehensiveness score, *RDS* resource disparity score

### Model results

According to Model 1 (Table 2), IPV-related health services are concentrated in areas with a larger population of individuals who are white, as compared to Hispanics and Other groups (*β* = .31, *Z* = 2.23, *p* = .026), or on social security benefits (*β* = .01, *Z* = 2.68, *P* = .007). In contrast, IPV resources are less concentrated in census tracts with a larger population of residents who are younger (*β* = −.02, *Z* = − 3.04, *P* = .007), low-income (*β* = −.19, *Z* = − 3.00, *P* = .003), and to a lesser extent, black, as compared to Hispanics and Other groups (*β* = −.18, *Z* = − 1.83, *P* = .067). Model 2 included all of the covariates from Model 1, but also included the IPV arrest rate. None of the significance levels for these relationships changed when the IPV arrest rate was included in Model 2. The IPV arrest rate was not significantly associated with NCS, thus revealing a spatial mismatch between IPV arrests and resource comprehensiveness.

**Table 2:** Spatial lag regression models of the mean normalized comprehensiveness score (NCS) on select sociodemographic characteristics (model 1), and on IPV arrest rate controlling for select sociodemographic characteristics (model 2) for 503 census tracts in Miami-Dade County

Characteristic	Model 1			Model 2		
	*β* (SE)	*Z*-score	*p*-value	*β* (SE)	*Z*-score	*P*-value
Constant	.73 (.22)	3.37	<.001	.73 (.22)	3.37	<.001
Black non-Hispanic (%)	−.18 (.10)	−1.83	.067	−.18 (.10)	−1.75	.081
White non-Hispanic (%)	.31 (.14)	2.23	.026	.31 (.14)	2.23	.026
Median Age (years)	−.02 (.01)	−3.04	.002	−.02 (.01)	−3.00	.003
Median Gross Rent ($1000s)	−.19 (.06)	−3.00	.003	−.19 (.06)	−2.99	.003
Social Security Benefits Recipients (%)	.01 (.00)	2.68	.007	.01 (.00)	2.66	.008
IPV Arrest Rate (per 1000)				−.00 (.00)	−.19	.846
Spatial Lag Term (Rho)	.91 (.02)	56.40	<.001	.91 (.02)	56.34	<.001
Model diagnostics	AIC = 788.49, *R*^2^ = .80			AIC = 790.45, *R*^2^ = .80		

*AIC* Akaike information criterion. % Hispanic/Other is reference category for race/ethnicity variable

Model 3 (Table 3) explored these spatial mismatches and identified five key predictors of the RDS. In a spatial error regression model, RDS was positively associated with percent white as compared to percent Hispanic/Other (*β* = 1.40, *Z* = 2.06, *P* = .039) and negatively associated with percent black as compared to percent Hispanic/Other (*β* = −.84, *Z* = − 1.98, *P* = .048), median age (*β* = −.07, *Z* = − 6.68, *p* < .001), ethnic heterogeneity (*β* = −.95, *Z* = − 2.01, *P* = .045), and concentrated disadvantage (*β* = −.35, *Z* = − 3.23, *P* = .001) after adjusting for the spatial error term. These findings indicated that black and economically disadvantaged areas were more likely to be under-resourced, while white areas were generally over-resourced.

**Table 3:** Spatial error regression model of the mean resource disparity score (RDS) on select sociodemographic characteristics for 503 census tracts in Miami-Dade County

Characteristic	*β* (SE)	*Z*-score	*P*-value
Constant	3.17 (.51)	6.16	<.001
Black non-Hispanic (%)	−.84 (.43)	−1.98	.048
White non-Hispanic (%)	1.40 (.68)	2.06	.039
Median Age (years)	−.07 (.01)	−6.68	<.001
Ethnic Heterogeneity Index	−.95 (.47)	−2.01	.045
Concentrated Disadvantage Index	−.35 (.11)	−3.23	.001
Spatial Error Term (Lambda)	.67 (.04)	17.15	<.001
Model diagnostics	AIC = 1536.87, *R*^2^ = .46		

*AIC* Akaike information criterion. % Hispanic/Other is reference category for race/ethnicity variable

**Fig. 2:**
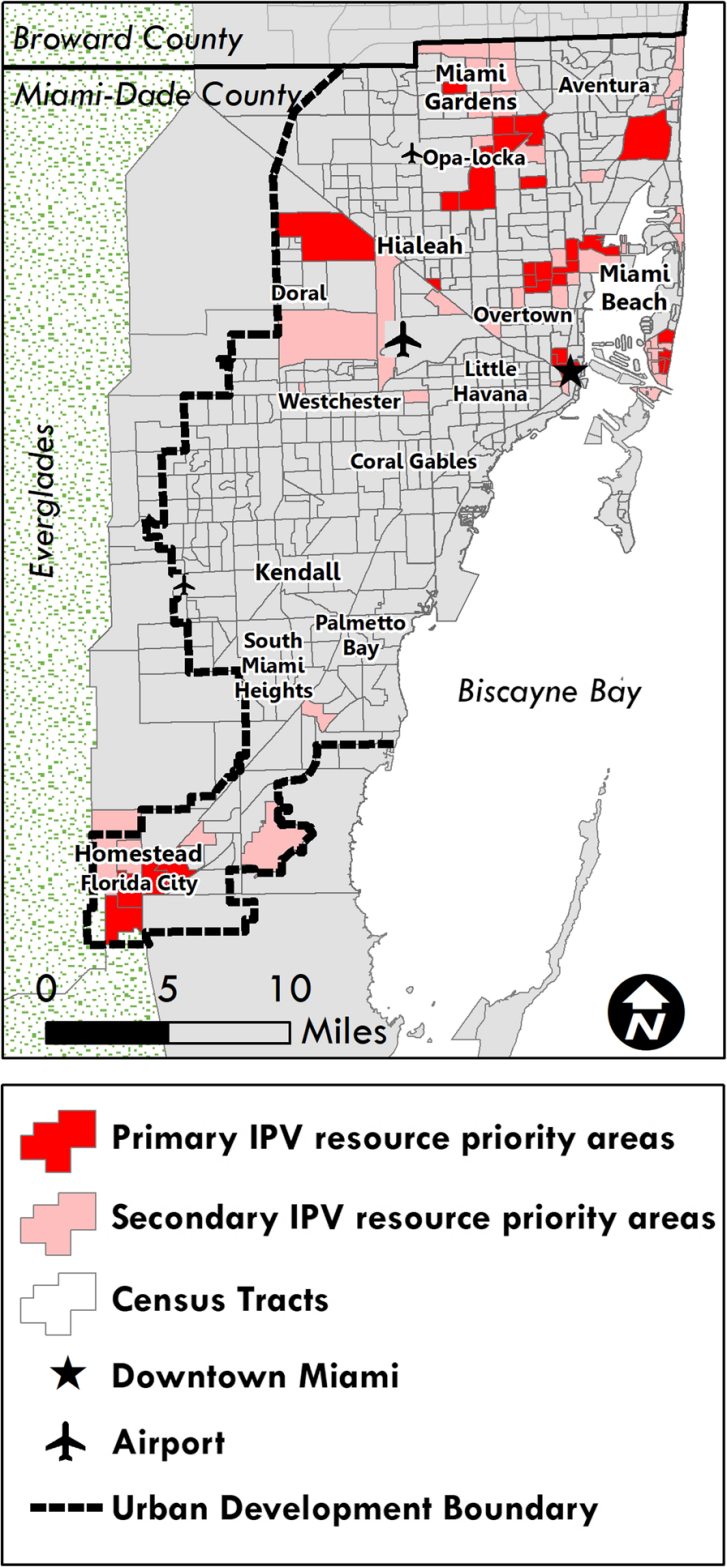
Census Tracts with Lowest Resource Disparity Score (RDS) and Highest Intimate Partner Violence (IPV) Arrest Rate. Census tracts falling into both the lowest quartile and decile of RDS, and the highest quartile and decile of IPV arrest rate. These tracts represent potential targets for IPV resource prioritization. *Source:* Figures generated by authors using ArcGIS 10.3 (ESRI, Redlands, CA)

Figure 2 displays areas with high rates of IPV that may also be under-resourced. Because tracts with the lowest RDS represent the most extreme disparity between IPV-related health resources and arrests, but not necessarily the highest IPV arrest rates, we queried tracts that are in the lowest quartile and decile of RDS as well as the highest quartile and decile of IPV arrest rates. These areas are concentrated in predominately black neighborhoods to the north and south, as well as in white areas in the north near Miami Beach and the Aventura area.

## Discussion

Based on prior work [21], we know that IPV-related health services are more concentrated in areas with higher proportions of white residents and those receiving social security benefits and less concentrated in areas with higher proportions of black, younger, and low-income residents. In the current study, we expanded on previous research by examining the role of IPV arrest rates in the availability of IPV-related health services. This study evaluated the degree of spatial mismatch between IPV-related health services and IPV arrest rates in MDC. We find that IPV arrest rates are not associated with the density of IPV-related health services, and factors associated with race and socio-economic class help explain the largest relative disparities between arrest rates and service availability. A GIS enabled us to visualize these disparities and identify communities that should be prioritized for IPV-related resources and interventions. These findings provided further insights about the geographic availability of IPV-related health services in MDC and community-level characteristics related to this availability. Our approach also demonstrates the utility of GIS for identifying potential priority regions for IPV prevention efforts and resource allocation not only for MDC but other regions as well.

Ideally, IPV-related health resources should be driven by the level of need within the local community. The matching of health resources based on community need is a mainstay of public health practice and more recently has been advocated for in clinical practice [37, 38]. Our findings, however, show this is not the case. A statistically significant relationship was not found between NCS and IPV arrest rates, indicating a mismatch between IPV-related healthcare services and local community needs. Several factors were found to be related to this mismatch. Specifically, our findings indicated that communities with higher percentage of black residents, younger residents, ethnic heterogeneity and economic disadvantage were more likely to be under-resourced with regards to IPV-related health services, and areas with a higher percentage of white residents tended to be over-resourced.

There are several possible explanations for this observed discrepancy in resource allocation and need. First, this may reflect a general lack of awareness among healthcare facilities as to the needs of local communities. The imbalance between individual-level clinical services and population health needs is well established [39, 40]. Increased efforts have been made to promote congruency in this area, such as provisions in the Patient Protection and Affordable Care Act (Pub. L., No. 111–148, 124 Stat. 119) requiring tax exempt hospitals to conduct community health needs assessment and implement strategies to meet identified needs. While several resources have been developed to assist hospitals in meeting this requirement [37, 38], our results indicate that more support is needed, especially for non-hospital clinics, which constituted the majority of facilities in this study, and often have limited resources compared with hospitals. In addition, health facilities located in economically disadvantaged neighborhoods are also affected by limited resources and competing demands [41, 42], which may prevent them from providing comprehensive IPV-related services. Finally, while responsibility for the delivery of healthcare services often falls on individual healthcare providers and facilities, it is important to acknowledge the complex system in which these services are delivered. The healthcare system is often driven by political and financial processes, which in turn shape and interact with the policies and practices of providers and facilities. Interventions designed to address these multilevel contributors are likely to be most successful in promoting equitable allocation of health care resources [43–45].

Finally, it is critical to consider racial/ethnic inequalities, particularly among women of color, when interpreting the results of this study. Our findings suggest that, in MDC, the provision of IPV-related healthcare services is not simply a function of need but is also influenced by race and class. These findings are consistent with overwhelming evidence that women of color are often most at-risk for health problems but are least able to access key health services [46–48], and data which show that black communities have disproportionately higher arrest rates [49], including for IPV [29]. Observed disparities among women of color are often the result of a complex intersection of inequalities and discrimination across multiple levels including race, sex, and class [50, 51].

As such, IPV resource disparities are important to address as they can further fuel racial, economic, and health inequalities at the individual and neighborhood levels. Failures to address the socio-health correlates of IPV by healthcare providers may contribute to the cycle of violence in minority neighborhoods that are already disadvantaged. Prior research indicates that crime, especially violent crime, can lead to further economic inequality at the neighborhood level through the displacement of residents and businesses [52]. In turn, the destabilization of low-income neighborhoods could actually increase crime in these areas given that concentrated economic disadvantage has been linked to increased IPV rates [23, 53]. In this way, disparities in IPV-related healthcare services not only have implications for the well-being of victims, but also for neighborhoods by perpetuating existing inequalities and contributing to high levels of IPV.

This study has important implications for future intervention work aimed at improving IPV-related services within healthcare facilities. By identifying disparities in service allocation and factors associated with these disparities, we can better target intervention efforts by prioritizing communities with the greatest need. Specifically, the priority census tracts for intervention would be those with the highest arrest rates and the lowest RDS, as depicted in Fig. 2. While these areas primarily coincide with black neighborhoods, some upper-income areas were identified in Miami Beach and the Aventura area, reminding us that the burden of IPV is not confined to any single demographic group.

There are several limitations of this study which should be considered when interpreting the results. While crime incident data is a commonly used community-level indicator of violence [54], prior research showing disparities in the reporting and policing of crime, including IPV, demonstrates that the IPV arrest rate is an imperfect proxy for IPV activity [29, 49]. As such, our findings may be driven, in part, by racial/ethnic disparities in arrests of IPV rather than actual incidence or prevalence of IPV in communities [29]. Like much of the prior research examining the geographic correlates of IPV, we also used census tracts as the unit of analysis. Tracts, however, may not be a meaningful scale for understanding IPV-related processes since they are designed for official census purposes and do not necessarily align with residents’ conceptions of their spatial surroundings. Individuals generally access healthcare services close to where they live, albeit with interaction of race and place [55]. But the geography of healthcare utilization is often more complex, as individuals may use services that are closer to work, shopping, or child care [56]. Utilization can also be shaped by social networks (e.g., in immigrant communities) [57] or social stigma [58], phenomena that operate at a range of scales. In addition, this study presented a cross-sectional analysis of data, which provides an observation at a single point of time. It may be that areas with access to comprehensive IPV-related healthcare services have successfully lowered IPV incidents over time and now appear to be over-resourced. A longitudinal assessment would enable us to examine these trends. Finally, this study focused on secondary prevention strategies delivered through healthcare facilities. Future research is needed examining geographic predictors of access to primary and tertiary prevention services for IPV, which have been historically limited [59–61].

## Conclusions

Critical health inequities exist in the U.S. based on race/ethnicity and socioeconomic class, with a widening of this gap often contributed to a lack of appropriate resources targeting those most in need [62]. To combat these disparities, new strategies are needed focused on improving prioritization of resources and better planning of resource allocation [63]. This study describes one such strategy, by identifying spatial mismatches between IPV-related healthcare services and arrests in MDC and demonstrating the utility of GIS for setting intervention priorities and allocating resources for IPV prevention efforts. Given the increasing competitiveness in obtaining funds to support IPV prevention work, innovative methods are critical as we continue work towards strengthening the role of the healthcare system in addressing IPV.

## Abbreviations

GIS: Geographic Information System
IPV: Intimate partner violence
MDC: Miami-Dade County
NCS: Normalized comprehensiveness score
RDS: Resource disparity score
